# Atherothrombosis model by silencing of protein C in APOE*3-Leiden.CETP transgenic mice

**DOI:** 10.1007/s11239-021-02488-2

**Published:** 2021-05-30

**Authors:** Yvonne K. Jongejan, Jeroen C. J. Eikenboom, Marion J. J. Gijbels, Jimmy F. P. Berbée, Bart J. M. van Vlijmen

**Affiliations:** 1 Department of Internal Medicine, Division of Thrombosis and Hemostasis, Leiden University Medical Center, P.O. Box 9600, 2300 RC Leiden, The Netherlands; 2 Einthoven Laboratory for Vascular and Regenerative Medicine, Leiden University Medical Center, Leiden, The Netherlands; 3 Department of Medical Biochemistry, Experimental Vascular Biology, Amsterdam UMC, University of Amsterdam, Amsterdam, The Netherlands; 4 Department of Molecular Genetics, Department of Pathology, Cardiovascular Research Institute Maastricht, GROW-School for Oncology and Developmental Biology, Maastricht University, Maastricht, The Netherlands; 5 Department of Internal Medicine, Division of Endocrinology, Leiden University Medical Center, Leiden, The Netherlands

**Keywords:** Apolipoprotein E3, Atherosclerosis, Mice, Protein C, Thrombosis

## Abstract

Murine atherosclerosis models are key for investigation of atherosclerosis pathophysiology and drug development. However, they do not feature spontaneous atherothrombosis as a final stage of atherosclerosis. Transgenic mice expressing both the human mutant apolipoprotein E form APOE*3-Leiden and human cholesteryl ester transfer protein (*CETP*), i.e. APOE*3-Leiden.CETP mice, feature a moderate hyperlipoproteinemia and atherosclerosis phenotype. In contrast to apolipoprotein E deficient (*Apoeˉ*^*/*^*ˉ*) mice, APOE*3-Leiden.CETP mice respond well to lipid-lowering and anti-atherosclerotic drugs. The aim of the study was to investigate whether silencing of anticoagulant Protein C (*Proc*) allows APOE*3-Leiden.CETP mice to feature thrombosis as a final stage of atherosclerosis. Female APOE*3-Leiden.CETP mice were fed a Western-type diet to induce advanced atherosclerosis, followed by an injection with a small interfering RNA targeting *Proc* (si*Proc*). Presence of atherosclerosis and atherothrombosis was determined by histologic analysis of the aortic root. Atherosclerosis severity in the aortic root area of APOE*3-Leiden.CETP mice varied from type “0” (no lesions) to type “V” lesions (advanced and complex lesions). Atherothrombosis following si*Proc* injection was observed for 4 out of 21 APOE*3-Leiden.CETP mice (19% incidence). The atherothrombosis presented as large, organized, fibrin- and leukocyte-rich thrombi on top of advanced (type “V”) atherosclerotic plaques in the aortic root. This atherothrombosis was comparable in appearance and incidence as previously reported for *Apoeˉ*^*/*^*ˉ* mice with a more severe atherosclerosis (19% incidence). APOE*3-Leiden.CETP mice with modest hyperlipidemia and atherosclerosis can develop atherothrombosis upon transient *Proc*-silencing. This further extends the use of these mice as a test model for lipid-lowering and anti-atherosclerotic drugs.

## Highlights

- APOE*3-Leiden.CETP mice with modest hyperlipidemia and atherosclerosis can develop atherothrombosis upon transient *Proc*-silencing.
- The featured atherothrombosis presented as large, organized, fibrin- and leukocyte-rich thrombi on top of advanced (type “V”) atherosclerotic plaques in the aortic root.
- The findings of this study further extends the use of APOE*3-Leiden.CETP mice as a test model for lipid-lowering and anti-atherosclerotic drugs.

## Introduction

Murine atherosclerosis models are widely used in studying atherosclerosis pathophysiology and development of novel therapeutic strategies. However, the available murine models do not feature the final stage of human atherosclerosis, that is, plaque rupture and/or erosion-induced atherothrombosis, underlying myocardial infarction and ischemic stroke[1–3]. Recently, we demonstrated for apolipoprotein E deficient (*Apoeˉ*^*/*^*ˉ*) mice with pre-existing advanced atherosclerosis that inhibition of anticoagulation by protein C (*Proc*)-silencing resulted in atherosclerosis-associated thrombus formation. This unique finding of ‘spontaneous’ atherothrombosis was at a low incidence and with reproducible histological appearance (19% of animals affected, three independent and controlled experiments)[2, 3]. A drawback of *Apoeˉ*^*/*^*ˉ* mice is their limited ability to subtly modulate their plasma cholesterol levels and extent of atherosclerosis. More importantly, these mice show poor response to established lipid-lowering and anti-atherosclerotic drugs, which limits their use as a (drug-)testing model[4]. In contrast, transgenic mice expressing both human mutant *APOE* form APOE*3-Leiden and human cholesteryl ester transfer protein (*CETP*) i.e. APOE*3-Leiden.CETP mice, feature a moderate and easy modifiable hyperlipoproteinemia and atherosclerosis phenotype[4, 5]. In addition, they respond well to lipid-lowering and anti-atherosclerotic therapeutics[4]. Here, we investigated whether *Proc*-silencing allows APOE*3-Leiden.CETP mice, like *Apoeˉ*^*/*^*ˉ* mice, to feature thrombosis as final stage of atherosclerosis.

## Methods

APOE*3-Leiden.CETP transgenic mice (C57BL/6J background) were bred and genotyped as previously described[5]. Female APOE*3-Leiden.CETP mice are more prone to develop advanced atherosclerosis and are more suitable for drug intervention studies and were therefore used. Four-week-old female mice were fed a semi-synthetic Western-type diet (WTD) containing 0.15% [w/w] cholesterol (AB diets, Woerden, The Netherlands) for 18 weeks to induce advanced atherosclerosis. At 4, 8 and 14 weeks on WTD, 50 µL EDTA blood was collected through tail bleeding, and plasma total cholesterol levels were determined (Roche Diagnostics, Mannheim, Germany). For the APOE*3-Leiden model (with or without human CETP transgene), the plasma cholesterol levels and duration of these levels (cholesterol exposure) are predictors of the extent of atherosclerosis[6]. Common to this model, to warrant atherosclerosis presence in the aortic root, only mice with plasma cholesterol levels above 7.5 mmol/L at all measured time points are included for small interfering (si)RNA injections[6]. After 18 weeks on WTD, hepatic *Proc* expression was silenced using a standardized protocol[2, 3, 7] in which mice were injected intravenously with synthetic siRNA (5 mg/kg, cat. #S72192, Ambion, Life Technologies, Carlsbad (CA), USA), complexed with Invivofectamine 3.0 (Life Technologies). The protocol proved to result in reproducible and effective *Proc*-lowering with limited variation in the level of knockdown among si*Proc*-treated animals[2, 3, 7]. Seven days post-injection, mice were anesthetized by subcutaneous injection of ketamine (100 mg/kg), xylazine (12.5 mg/kg) and atropine (125 µg/kg), and subsequently euthanized by exsanguination through disruption of aorta and caudal vena cava, followed by isolation and formalin-fixation of heart and descending aorta. All experimental animal procedures were according to (inter)national guidelines (Directive 2010/63/EU). After paraffin-embedding, serial sections (5 µm) of the aortic root were prepared, stained with hematoxylin and eosin (HE), and used for atherosclerosis and atherothrombosis analyses[8]. Based on the findings in *Apoeˉ*^*/*^*ˉ* mice, we confined analysis to the aortic root and atrium, not the descending aorta[2]. These analyses included measurements of total lesion area and individual plaque size for all mice, starting from the appearance of the open aortic valve leaflets in four subsequent sections with 50-µm intervals, and cap thickness and necrotic core size for size-matched plaques with and without atherothrombosis using ImageJ software (version 1.53e). For some comparison with the *Apoeˉ*^*/*^*ˉ* model, we re-analyzed HE-stained cryosections of the aortic root from female *Apoeˉ*^*/*^*ˉ* mice treated with si*Proc* that were generated in a previous study[3]. Descriptive statistical analysis (Wilson-score) on thrombosis incidence was performed using OpenEpi (http://openepi.com/Proportion/Proportion.htm). A two-tailed Mann Whitney U test was performed for comparing mice and plaques with and without atherothrombosis within the APOE*3-Leiden.CETP group (GraphPad Prism version 8.4.2, GraphPad Software, San Diego, CA, USA), *P* < 0.05 was considered to be statistically significant.

## Results and discussion

**Fig. 1:**
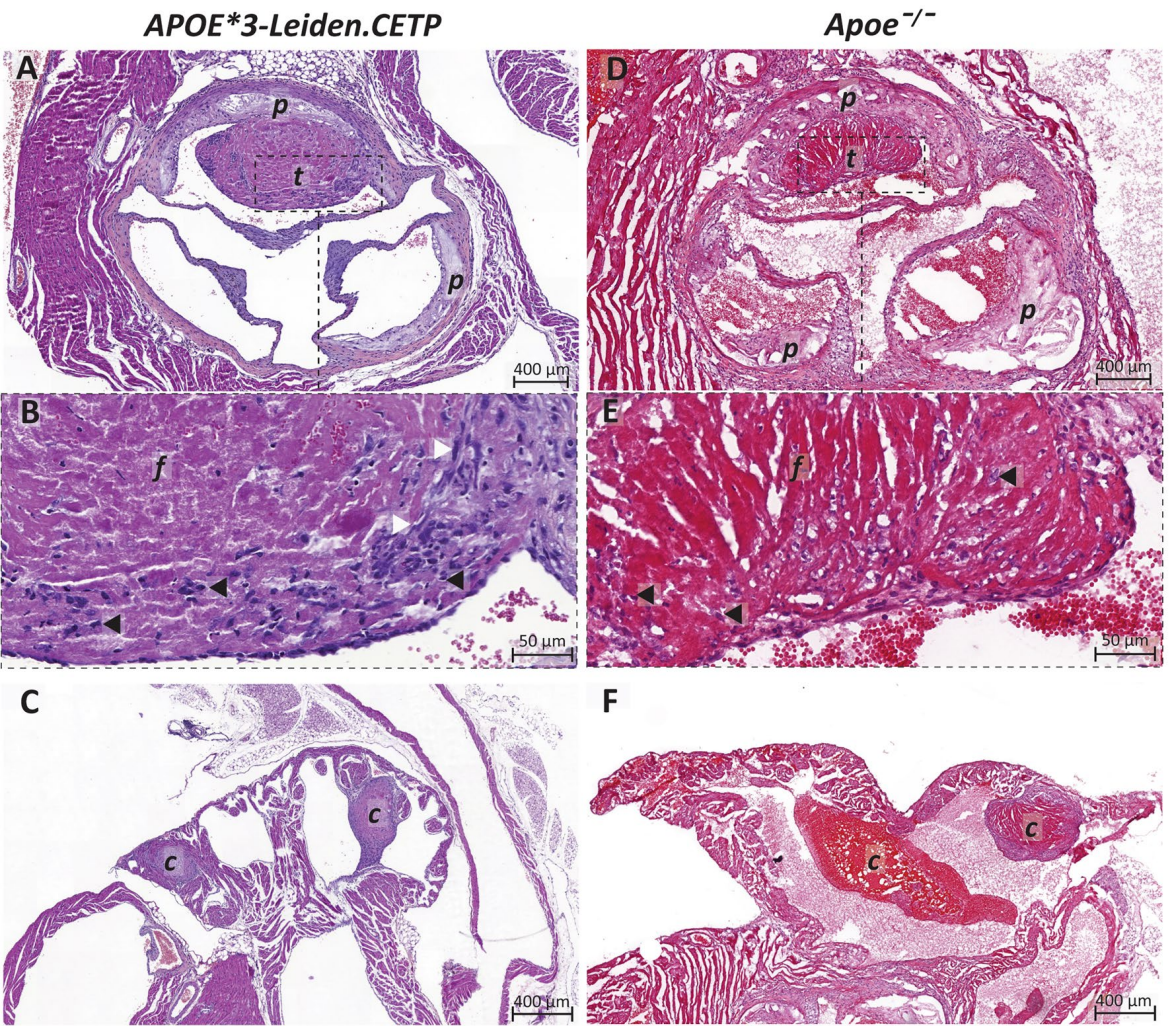
Atherothrombosis following si*Proc*-treatment in atherosclerotic APOE*3-Leiden.CETP and *Apoe*ˉ^/^ˉ mice. **A** Representative image of HE-stained paraffin aortic root section with atherothrombosis (*t*) associated with an advanced atherosclerotic plaque (*p*) in APOE*3-Leiden.CETP mice. **B** Zoomed-in atherothrombus showing structures morphologically identified as fibrin (*f*), leukocytes (black arrowhead) and fibroblasts (white arrowhead). **C** Atrium with clot (*c*) presence. For comparison, representative images of atherothrombosis (**D, E**) and atrial clotting (**F**) in *Apoeˉ*^*/*^*ˉ* mice. Note: *Apoeˉ*^*/*^*ˉ* mice images are from previously generated HE-stained cryosections[3]

Following WTD feeding, 21 out of 27 APOE*3-Leiden.CETP mice had consistent plasma cholesterol levels above 7.5 mmol/L (see methods), and received si*Proc*-treatment. None of the si*Proc*-treated mice showed any abnormalities in appearance or behavior up to the moment of sacrifice. All treated APOE*3-Leiden.CETP mice developed atherosclerosis in the aortic root area typical to these mice, and ranged from valve segments without lesions (“type 0”) to presence of advanced atherosclerotic plaques (“type V”, Table 1)[8]. For 4 of the 21 hypercholesterolemic si*Proc*-treated APOE*3-Leiden.CETP mice (19%) we detected thrombus formation on top of an advanced atherosclerotic plaque. As morphologically identified from the HE-staining, these thrombi were presented as large, organized, fibrin- and leukocyte-rich with fibroblast presence (Fig. 1A, B) and were virtually identical to atherothrombi previously observed in *Apoeˉ*^*/*^*ˉ* mice, however for *Apoeˉ*^*/*^*ˉ* mice, thrombi were not as organized (Fig. 1D, E [2, 3]). Although anticipated for, the comparable atherothrombosis incidence for APOE*3-Leiden.CETP and *Apoeˉ*^*/*^*ˉ* mice is remarkable, considering the on average lower plasma cholesterol levels and less severe atherosclerosis in APOE*3-Leiden.CETP mice (Table 1, Fig. 1A, D). However, one should note that still 51% of the valve segments in the (21) APOE*3-Leiden.CETP mice have advanced atherosclerotic plaques (“type V” lesions, Table 1). The advanced plaques are a prerequisite for the atherothrombosis upon *Proc-*silencing, as shown here and in the earlier studies[2, 3]. The median [lower–upper range] total lesion area for mice with atherothrombosis was 336 [276–358] × 10^3^µm^2^ (n = 4) and for mice without atherothrombosis 239 [83–461] × 10^3^µm^2^ (n = 21) and did not reach statistical significance (*P* = 0.065). Regarding the individual plaques, the size of the plaques associated with atherothrombosis was 133 [103–198] × 10^3^µm^2^ (n = 4) and distributed to the high end of what was observed for plaques without atherothrombosis with 73 [0–228] × 10^3^µm^2^ (n = 57), although this did not reach statistical significance (*P* = 0.063). Cap thickness and necrotic core size were determined as markers for plaque stability. However, the difference in cap thickness between plaques with (23 [6–44] µm) and without atherothrombosis (28 [0.9–54] µm) was not statistically significant (*P* = 0.81), and neither was the necrotic core size (*P* = 0.57) for plaques with (25%, [19–35%]) and without atherothrombosis (20.4% [13–57%]). In addition, thrombosis-associated plaques were without signs of disrupted fibrous caps and/or intraplaque bleeding, from which we speculate that the atherothrombosis could originate from plaque erosion rather than plaque rupture. These findings are in line with our previous findings for *Apoeˉ*^*/*^*ˉ* mice treated with si*Proc*[2, 3]. For these *Apoeˉ*^*/*^*ˉ* mice, we previously demonstrated in three independent experiments, using a negative control siRNA injection (n = 33) compared with si*Proc*-injected (n = 68) animals, that the atherothrombosis solely follow from *Proc*-silencing. Previously, we found for *Apoeˉ*^*/*^*ˉ* mice that the atherothrombosis had a predilection for the right coronary sinus of the aortic root[2, 3], this could not be confirmed for APOE*3-Leiden.CETP mice. However, the relatively small animal numbers may underlie absence of such confirmation.

**Table 1:** Plasma cholesterol levels, lesion type and incidence of atherothrombosis in si*Proc*-treated APOE*3-Leiden.CETP (n = 21) and si*Proc*-treated *Apoeˉ*^*/*^*ˉ* (n = 25^a^) mice

	APOE*3-Leiden.CETP	*Apoeˉ*^*/*^*ˉ*^a^
Plasma cholesterol (mmol/L)	13.8 [7.6–19.0]	30.2 [12.2–51.0]
Lesion type (%)		
0/I/II/III/IV/V	6/6/3/10/24/51	0/0/0/0/0/100
Incidence (proportion; [range])		
Atherothrombosis	4/21 (19%; [7.7–40])	13/68^b^ (19%; [11.5–30.0])
Atrial clotting	7/21 (33%; [17.2–54.6])	7/25 (28%; [14.3–47.6])

^a^To compare with the *Apoeˉ*^*/*^*ˉ* model, from a previous study[3], data were included and materials re-analyzed

^b^The presented atherothrombosis incidence in *Apoeˉ*^*/*^*ˉ* mice is from 3 independent experiments from previous studies[2, 3]

7 out of 21 (33%) APOE*3-Leiden.CETP mice also featured signs of clotting in at least one atrium of the heart (Fig. 1C). Among these mice, one also developed atherothrombosis. While atrial clotting was also observed in *Apoeˉ*^*/*^*ˉ* mice (28%), the appearance of these atrial clots in APOE*3-Leiden.CETP mice was consistently organized and in two mice found in both atria. In contrast, only 1 out of 7 *Apoeˉ*^*/*^*ˉ* mice with atrial clotting featured an organized clot and all were confined to the left atrium (Fig. 1F [3]). The presence of atrial clotting indicates that surfaces other than atherosclerotic plaques trigger coagulation particularly in (arterial) areas of low flow and shear, as present in atria and around aortic valves. In this respect, it is important to denote that upon *Proc*-silencing wild-type C57BL/6J mice, as well as the venous system in *Apoeˉ*^*/*^*ˉ* mice, are free of clotting[9].

Altogether, we demonstrate that APOE*3-Leiden.CETP mice with modest hyperlipidemia and atherosclerosis can develop atherothrombosis upon transient *Proc*-silencing, with a presentation that is virtually identical to the atherothrombosis found in *Apoeˉ*^*/*^*ˉ* mice. The finding of atherothrombosis in APOE*3-Leiden.CETP mice further extends the use of these mice as a test model for lipid-lowering and anti-atherosclerotic drugs, with atherothrombosis resulting from *Proc*-silencing as an end-point challenge after prolonged exposure of mice to the drug treatment.
